# TE studies in Japan: The third Japanese meeting on host-transposon interactions

**DOI:** 10.1186/s13100-016-0082-8

**Published:** 2016-12-07

**Authors:** Kenji Ichiyanagi

**Affiliations:** Laboratory of Genome and Epigenome Dynamics, Department of Applied Molecular Biosciences, Graduate School of Bioagricultural Sciences, Nagoya University, Furo-cho, Chikusa-ku, Nagoya, 464-8601 Japan

**Keywords:** Transposable elements, Host defense, Exaptation, Disease, Cis elements

## Abstract

The third Japanese meeting entitled “Biological Function and Evolution through Interactions between Hosts and Transposable Elements (TEs)” was held on 5–6 September 2016 at National Institute of Genetics (NIG), Mishima, Japan. Supported by NIG, the goal of the meeting was to bring together researchers who study diverse biological phenomena such as schizophrenia, carcinogenesis, cellular reprograming, skin function, placental formation, plant mutagenesis and epigenetics, and small RNA-mediated heterochromatinization, where TEs are involved in various ways. The meeting included 13 invited speakers. Here we present highlights of these invited talks.

## Introduction

Transposable elements (TEs) are very important parts of genomes, as they play various roles in biological functions and evolution. Their important roles were evident at the third Japanese meeting entitled “Biological Function and Evolution through Interactions between Hosts and Transposable Elements (TEs)” which was held on 5–6 September 2016 at National Institute of Genetics (NIG), Mishima, Japan, with a beautiful view of Mt. Fuji. Before these TE meetings, the first of which was held in 2013, there were very few opportunities in Japan for TE researchers to gather to exchange their ideas. For the third meeting in September, we had 13 invited speakers in six sessions chaired by Kei Fukuda (RIKEN institute, Japan), Hidenori Nishihara (Tokyo Institute of Technology, Japan), Toshiki Habu (National Institute of Agricultural Science, Japan), Hidetaka Ito (Hokkaido University, Japan), Hidetoshi Saze (Okinawa Institute of Science and Technology, Japan), and the author, Kenji Ichiyanagi (Nagoya University, Japan). There were many questions and comments from the more than 50 attendees and from the session chairs with time allowed for extensive and profound discussions.

## Highlights of the talks

### TEs and the nervous system

Right after the opening remarks by Ichiyanagi, Yukihito Ishizaka (National Center for Global Health and Medicine, Japan) presented an interesting link between HIV infection, LINE-1 (L1) retrotransposition, and neurocognitive disorders in humans. He and his colleagues found that L1 mobility is increased in 6 of 15 blood samples of HIV-1 positive patients, and that the Vpr protein of HIV-1 is responsible for the induction of L1 retrotransposition [1]. Moreover, in mice, intraperitoneal injection of recombinant Vpr (rVpr) induces L1 retrotransposition in the hippocampus, which is associated with impaired memory recall. Importantly, this rVpr-induced cognitive disorder is blocked by co-administration of Stavudine, an inhibitor of reverse transcriptase, suggesting that L1 retrotransposition plays a role in the pathogenesis. The rVpr-injected mice have increased expression of type I interferon, glutaminase C, glutamate and p17 subunit of Caspase 3, whereas rVpr-injected *Irf7*-knockout (KO) mice do not, suggesting an involvement of the interferron-dependent cellular cascade [2]. Taken together, environmental factors (Vpr in blood, in this case) can induce L1 retrotrotransposition in somatic cells, which results in the development of pathological conditions [3]. Miki Bundo (Kumamoto University, Japan) presented a link between schizophrenia, a psychiatric disorder, and L1 retrotranspsition in neurons. By qPCR-based L1 quantification for neurons purified from postmortem prefrontal cortex, she and her colleague revealed that the L1 copy number is increased in patients with schizophrenia [4]. Interestingly, *de novo* L1 insertions preferentially reside around genes important for synaptic function, suggesting an involvement of these L1 insertions in pathogenesis of schizophrenia. L1 amplification in the prefrontal cortex is also observed for animal models when the immune system is activated (poly I:C dsRNA injection in mice and EGF treatment of macaques). Again, environmental factors induce L1 retrotransposition in somatic cells. She also presented data showing that L1 is amplified in neurons derived from induced pluripotent stem cells (iPSCs) from patients with the *22q11.2* deletion syndrome with schizophrenia. Thus, genetic factors also play a role. Identifying the type and location of the neurons that accumulate *de novo* L1 insertions in the brain of schizophrenia patients, which is now feasible by single cell genomics techniques, will provide further insights on the relationship between L1 retrotransposition and psychiatric disorders.

### ERVs and gene expression

Evidence has been accumulating that endogenous retroviruses (ERVs) can serve as an enhancer or promoter of host genes, and their expression patterns are likely programmed by a combination of host transcription factors. Kosuke Hashimoto (RIKEN institute, Japan) presented his recent results about a transcriptome study of c-Myc-induced liver tumors, which reveals that transcription from LTR sequences of several mouse ERV families are increased by up to 1000-fold, a situation similar to those in virus-induced tumors [5]. Shutting off c-Myc expression results in downregulation of these ERVs back to background levels, and ChIP-seq data shows direct binding of the c-Myc protein to these LTRs. In normal mice, the expression of these ERVs is high in embryonic tissues, then gradually declines along with embryonic growth, reaching the background level after birth. Interestingly, host genes that are upregulated by c-Myc show a very similar trajectory of expression dynamics during embryonic development. Thus, the expression of these ERVs as well as “embryonic genes” is developmentally programmed, and can be re-activated by c-Myc upon tumorigenesis. Mari Ohnuki (Ludwig Maximilians University Munich, Germany), a former post-doc in Shinya Yamanaka’s lab at Kyoto University, presented a talk about functional involvement of an ERV in reprograming of somatic cells. Although iPSCs are characterized by universal differentiation potential and limitless proliferation, such abilities are considerably variable across cell lines. She and her colleague screened for iPSCs that are defective in differentiation into neuronal cells, and found that the isolated defective iPSCs express higher levels of long non-coding RNAs (lncRNAs) that are transcribed from an upstream LTR7 sequence [6]. This is not simple coincidence but is functionally important, because transfection of shRNA against the LTR7 sequence suppresses the differentiation defects of these iPSCs [7]. The LTR7 elements themselves (which are the LTRs of HERVH) are also expressed in iPSCs, embryonic stem cells (ESCs), and epiblasts, and their expression is directly induced by pluripotency-related transcription factors, POU5F1 (also known as OCT4), SOX2 and KLF4 [7]. Ancestral viruses of HERVH/LTR7 have invaded the primate genomes several times during evolution, generating species-specific HERVH/LTR7 insertions. Interestingly, such species-specific insertions are associated with species-specific gene expression profiles observed in human and macaque iPSCs. Thus, an intriguing idea is that HERVH/LTR7 insertions have contributed to the diversification of the pluripotency-related regulatory network in the course of primate evolution.

### TE exaptation

Examples of TE exaptation, where TEs give rise to functional sequences, are not restricted to DNA segments. TE-encoded proteins can also be exapted. Takeshi Matsui (RIKEN-IMS, Japan) talked about a protease derived from the *pro* region of an ERV, termed SASPase (Skin Aspartic Protease), which is expressed in epidermis. For terrestrial vertebrates, the stratum corneum, the uppermost dead cell layer of epidermis constituting the boundary between the body and environment, is crucial to adapt and live in a dry environment. In the stratum corneum of mammals, which have supple and moisturized skin, the profilaggrin protein is hydrolyzed to yield filaggrin monomers, which play a role in barrier function of the stratum corneum and are a major predisposing factor for atopic dermatitis. Interestingly, SASPase is conserved among mammals, and moreover, is involved in profilaggrin hydrolysis. KO mice of this ERV-derived protease show a dry-skin phenotype due to abnormal proteolysis of profilagrin and consequent reduction of filagrin [8]. Thus, exaptation of this protease is involved in the acquisition of the mammalian-specific soft and moisturized skin for sensitive perception of external environmental changes. Takayuki Miyazawa (Kyoto University, Japan) talked about ERV-derived proteins working as another type of barrier. In humans, Syncytine-1, which is derived from the envelope protein of HERVW, plays a role in cell fusion in the syncytiotrophoblast in placenta. The placenta also expresses another ERV-derived protein, termed Suppressyn, which originates from HERV-Fb1. Suppressyn suppresses the cell fusion function of Syncytine-1 by inhibiting the interaction between Syncytine-1 and the receptor, ACST-2. Interestingly, Suppressyn is present in macaque and baboon species, which have already lost Syncytine-1, suggesting that Suppressyn has a role other than Syncytine-1 inhibition. It was then shown that Suppressyn acts in the host defense system: Supressin can protect the host from infection by other retroviruses such as Simian retrovirus 2 (SRV-2), SRV-4, BaEV, and RD-114 [9]. Fumitoshi Ishino (Tokyo Medical and Dental University, Japan) gave a talk about multiple exaptation events, giving a gene family of *Sushi-ichi-related retrotransposon homologues* (*SIRHs*). Among 11 *SIRH* genes in mammals, he and his colleague have shown that *Peg10* (also known as *Sirh1*), *Peg11* (*Sirh2*/*Rtl1*) and *Sirh7 (Ldoc1)* are essential for development and reproduction, and implicated in multiple aspects of placental function [10–12]. In addition to these, he presented data where *Sirh11* (*Zcchc16*) KO mice exhibit various behavioral abnormalities, possibly related to the noradrenergic system [13]. Interestingly, *Sirh11* is retained in three groups of the eutherians (euarchontoglires, laurasiatheria and afrotheria), whereas it is heavily mutated in xenarthrans such as armadillo. Moreover, a large variation in the SIRH11 protein is detected even in the three groups in a lineage-specific manner; for example, a loss of a C-terminal CCHC RNA-binding domain that occurred independently in gibbons and megabats and a loss of the N-terminal half that occurred independently in New World monkeys, Hystricognaths (such as Porcupine), and Cetartiodactyla (whales and ruminants). Therefore, *SIRH11* may have contributed to the diversification of eutherians by lineage-specific structural changes and subsequent species-specific functional changes, which have been fixed via complex natural selection events [13].

### Deleterious and advantageous effects of TE mobility

Of course, TE mobility often confers deleterious effects rather than benefits. Kazuo Tsugane (National Institute for Basic Biology, Japan) presented molecular mechanisms by which active TE transposition affects the host phenotype. In rice, *nDart1,* a non-autonomous element associated with a hAT family of DNA transposon *aDart1*, has generated various mutants under natural growth conditions due to its tendency to insert into genic regions [14]. Among them, a recessive mutant, named *snow-white leaf1* (*swl1*), shows variegated albino phenotype. He and his colleague revealed that it is caused by an insertion of *nDart1* in the SWL1 gene, which was subsequently revealed to be essential for thylakoid membrane organization during chloroplast development. The *nDart1* insertion inhibits the production of the wild-type protein by interfering with transcription from the normal start site. Of interest is that this albino phenotype is variegated. This is because the inserted copy sometimes gets excised by the *aDart1* transposase during somatic growth [15]. Akira Kanazawa (Hokkaido University, Japan) also presented an example of gene disruption by a TE insertion, but in this case, the disrupted gene shows an interesting evolutionary fate. Soybean, a paleopolyploid plant, contains a large number of duplicated genes. He and his colleague isolated two functionally redundant paralogs, *GmphyA1* and *GmphyA2*, which encode a photoreceptor, phytochrome A (phyA). They then characterized these genes in both photoperiod-sensitive and insensitive soybean lines [16]. In the photoperiod-insensitive lines, the *GmphyA2* gene is disrupted by an insertion of a novel *Ty1/copia*-like retrotransposon named *SORE-1* (Soybean Retroelement 1) into the coding region, and this *SORE-1* insertion causes photoperiod insensitivity. Interestingly, the *SORE-1*-inserted allele of *GmphyA2* is present only in soybean lines grown in high latitude regions of Japan [17]. Why is such an allele selected in these populations? The photoperiod insensitivity causes early plant maturation, and this trait is advantageous in the high latitude regions where the crop season is restricted. Thus, the *GmphyA2* disruption by the *SORE-1* insertion led to adaptation to a particular environment, while the buffering effect of gene duplication has sustained the plants, a novel consequence of nonfunctionalization of duplicate genes by TE insertion [17]. In view of agricultural application, *SORE-1* is a potentially useful mutagen that induces heritable mutations, because its transcription occurs during the reproductive phase. In addition, *SORE-1* appears to be controlled by an epigenetic mechanism involving small RNAs. Thus, artificial control of its epigenetic state may provide a tool to engineer novel traits in soybean.

### The host-TE arm race

Despite many TE exaptation events, the host-TE interactions present some sorts of arm race, because of the potential harmfulness of TE mobility. For example, TEs are usually transcriptionally silenced by epigenetic mechanisms while a subfamily can emerge that escapes such regulation. Aoi Hosaka (National Institute of Genetics, Japan), a graduate student in Tetsuji Kakutani’s lab, talked about a TE family that combats epigenetic silencing mechanisms. The *Arabidopsis* transposon VANDAL21 is silent in wild-type plants due to heavy DNA methylation [18]. However, when *VANC21*, one of three VANDAL21-encoded genes, is introduced into the wild-type background as a transgene, it reduces the DNA methylation levels of the *endogenous* copies of VANDAL21, resulting in their active transcription and mobilization [19]. These data indicate that the VANC21 protein functions as an anti-silencing factor. Interestingly, this anti-silencing effect by VANC21 is highly specific to VANDAL21 copies, because other TEs, including other closely related VANDAL family members, are not affected. This sequence-specific, rather than global, anti-silencing activity seems a good strategy for the TE to proliferate with minimizing deleterious effects to the host [19]. They also analyzed genomic regions where VANC21 binds, which identified several sequence motifs and provided insights on the molecular basis for the rapid evolution of this anti-silencing system.

### RNAi and chromatin regulation

In addition to DNA and histone modifications, small RNAs constitute an important part of the host defense system. Although post-transcriptional gene silencing (PTGS) is a built-in activity for small RNA-mediated regulation, small RNAs are also involved in transcriptional gene silencing (TGS) in many cases. Kuniaki Saito (Keio University, Japan), who has been studying the mechanism of transcriptional repression mediated by Piwi-interacting RNAs (piRNAs) in the *Drosophila* ovarian somatic cell line (OSC), presented his recent results that revealed that the Piwi protein physically associates with linker histone H1. Indeed, depletion of Piwi decreases the histone H1 density, as well as the heterochromatin protein 1a (HP1a) density, on target loci [20]. Loss of H1 results in the increase in RNA Polymerase II (RNAPII) occupancy and chromatin accessibility at target loci without affecting H3K9me3 and HP1a density. Moreover, knockdown of both H1 and HP1a synergistically increased TE expression in OSCs. Therefore, the Piwi-mediated TE silencing in *Drosophila* ovary likely consists of two pathways; (1) modulation of chromatin accessibility by regulating H1 density and (2) establishment and maintenance of heterochromatin by recruiting HP1a.

Like in flies, piRNAs of 26–30-nucleotide (nt) are generated in male and female germ cells (but not in gonadal somatic cells) in mice. The piRNA pathway in mouse male germ cells is essential for spermatogenesis and repression of some families of retrotransposons. In oocytes, however, the regulation and function of piRNAs are poorly understood. Yuka Kabayama (Kyushu University, Japan), a graduate student in Hiroyuki Sasaki’s lab, investigated the consequences of loss of piRNA-pathway components in oocytes. She showed that a KO mutation of *Mili* (also known as *Piwil2*) causes an almost complete loss of oocyte piRNAs, which results in an increase in transcripts from some specific retrotransposons, similar to what has been observed in male germ cells [21, 22]. On the other hand, a KO mutation of *Pld6* (also known as *mZuc* or *Mitopld*), encoding a mitochondrial nuclease/phospholipase, decreases the level of piRNAs to only a half of that in wild-type oocytes, which is in stark contrast to the situation in males where the same mutation severely affects piRNA biogenesis [23]. Because the PLD6 protein cleaves piRNA precursors to generate primary piRNAs in males [24], these results suggest the presence of other nuclease(s) in oocytes. Interestingly, they identified novel small RNAs of 21- to 23-nt in length derived from retrotransposons. These RNAs show the 10-nt complementarity with 26- to 30-nt piRNAs, suggesting that they are produced via the ping-pong cycle catalyzed by MILI. Indeed, the production of the 21- to 23-nt RNAs is dependent on MILI but independent of DICER. While the function of these shorter RNAs remains to be elucidated, this study highlights that species, biogenesis, and function of piRNAs differ between sexes in mice.

In fission yeast, heterochromatin is formed and maintained via a small RNA pathway, and this system continues to provide mechanistic insights of small RNA-mediated heterochromatinization. Yota Murakami (Hokkaido University, Japan) presented a mechanistic link between phosphorylation of RNAPII and efficiency of small RNA-mediated heterochromatinization. Previously, he and his colleague revealed that RNAPII is important for siRNA generation and heterochromatinization of the centromeres [25]. The C-terminal domain (CTD) of RNAPII is composed of repeats of Tyr1-Ser2-Pro3-Thr4-Ser5-Pro6-Ser7, and phosphorylation of different serine residues regulates different steps of transcription, which is called the CTD code. At this time, he showed that Ser7 of the RNAPII CTD is required for efficient siRNA generation and heterochromatin formation in fission yeast. Interestingly, Ser7 facilitates chromatin retention of nascent heterochromatic ncRNAs (hncRNAs), which directs efficient generation of siRNAs. Chromatin retention of hncRNAs, and siRNA generation as well require an RNA-binding activity of the chromodomain of Chp1, a subunit of the RNA-induced transcriptional silencing (RITS) complex, which contains siRNA and an Argonaute homologue, Ago1. This RITS complex associates with RNAPII in a Ser7-dependent manner, meaning that Ser7 plays a pivotal role in RNA-induced hetrochromatinization through its phosphorylation to recruit the RNA-chromatin connector protein, Chp1. This finding reveals a novel function of the CTD code: linking ncRNA transcription to *cis*-acting RNAi.

## Concluding remarks

The meeting was fruitful: the participants enjoyed it, and they exchanged their recent results and ideas throughout the meeting including coffee breaks and banquet. Three months before this meeting, an international TE meeting (1st Korea-Japan International Symposium for Transposable Elements) was held in Busan, Korea, which was organized by Dr. Heui-Soo Kim (Pusan National University, Korea) and supported by the Korean Society for Molecular and Cellular Biology. The second international TE meeting is now being planned (chief organizer: Dr. Fumitoshi Ishino), which will be held in Tokyo in 2017. There will be researchers not only from Japan and Korea, but also from other countries in Asia, which will enhance and extend TE research in Asia.
